# Efficacy of Original Neurofeedback Treatment Method for Brain Fog From COVID-19: A Case Report

**DOI:** 10.7759/cureus.56519

**Published:** 2024-03-20

**Authors:** Tatsuya Masuko, Harue Sasai-Masuko

**Affiliations:** 1 Department of Orthopedic Surgery, Emerald Orthopedic & Pain Clinic, Sapporo, JPN

**Keywords:** mild to severe cognitive dysfunction, z-score, neurofeedback therapy, electroencephalography (eeg), brain fog, covid-19

## Abstract

Brain fog is one of the most well-known sequelae of long COVID. It causes cognitive problems, mostly short-term memory disturbances, attention impairments, and problems with concentration. Although trials for treatment methods for brain fog have been carried out worldwide, effective methods have not yet been reported. Neurofeedback is effective for several common disorders and symptoms, including anxiety, depression, headaches, and pain. Neurofeedback is also reported to improve cognitive functions, such as processing speed and executive functions, including attention, planning, organization, problem-solving, and performance. Furthermore, neurofeedback is effective for “chemofog” and “chemobrain,” which occur after chemotherapy and cause cognitive impairments in a similar manner to brain fog. However, there have been no reports of neurofeedback treatments for brain fog. Therefore, we have started to develop an original neurofeedback treatment method for brain fog using a Z-score neurofeedback technique. In this study, we present the first case report of a patient who has successfully recovered from brain fog via neurofeedback. Pain and psychological assessments revealed that the patient’s pain improved and that the patient recovered from anxiety. Electroencephalograph data revealed several noble findings. C4 was thought to be the most affected site by brain fog, and this improved after treatment. The percentage increase at alpha wavelengths increased at almost all sites, and beta 1, beta 2, beta 3, and Hi beta decreased at almost all sites. The increased values at theta and alpha wavelengths after the 1st and 2nd sessions and the decreased values at higher beta wavelengths, such as beta 3 and Hi beta, were shown at all sessions.

## Introduction

Long COVID or post-COVID-19 condition (PCC) describes the long-term complications after acute COVID-19 infection, and it is the affection of many organs such as the heart, brain, kidney, pancreas, and digestive system. The clinical symptoms of long COVID are reported as fatigue, dyspnea, myalgia, diffuse pain, headaches, anxiety or depression, and cognitive impairments [1]. These long-lasting disabilities cause serious problems for daily activities.

Brain fog is one of the most well-known sequelae of long COVID and causes cognitive problems, mostly short-term memory disturbances, attention impairments, and problems with concentration [2]. The pathophysiology of brain fog is reported to be due to direct viral neuronal damage, neuroinflammation, rupture of the blood-brain barrier, microvasculitis, and hypoxia [1]. The occurrence of brain fog is thought to relate to chronic inflammatory processes in the nervous system and disturbed neurotransmission. A study based on fluorodeoxyglucose-positron emission tomography (FDG-PET) reported that patients who suffered from long COVID had hypometabolic regions of the cingulate cortex.

Neurofeedback is effective for several common disorders and symptoms, including anxiety, depression, insomnia, headaches, and pain [3], and it can change brain connectivity, such as the activity of the default mode network (DMN) (overactivity of the DMN is thought to be one cause of anxiety). Neurofeedback can also improve cognitive functions such as processing speed and executive functions, including attention, planning, organization, problem-solving, and performance [4].

More than 50% of patients who undergo chemotherapy develop symptoms similar to those described above for long COVID [5]; this condition is called “chemofog” or “chemobrain.” The left inferior frontal cortex, superior and middle frontal gyri, parahippocampal gyrus, cingulated gyrus, and precuneus are affected by chemotherapy [6]. Neurofeedback is effective for “chemofog” and “chemobrain” [7]. Self-reported cognitive function, fatigue, sleep quality, and psychological well-being are significantly improved using neurofeedback [7]. Based on these findings on “chemofog” and “chemobrain,” the efficacy of neurofeedback treatment for brain fog is highly promising.

There have been no reports of neurofeedback treatment for brain fog. Therefore, we have started to develop an original treatment method for brain fog using neurofeedback. In this study, we present the first case report of a patient who has successfully recovered from brain fog caused by COVID-19 infection using our original method, “two-by-four 8-channel Z-score neurofeedback treatment for brain fog.”

## Case presentation

A 34-year-old right-handed female in good health was diagnosed with acute COVID-19 infection based on the positive result of a polymerase chain reaction (PCR) test. She had no risk factors such as obesity, diabetes, or hypertension. After a fever that lasted for only one day, she developed a variety of symptoms such as general fatigue, cough, breathlessness, smell disorder, and taste disorder. Although we did not understand why another physician prescribed escitalopram oxalate, a selective serotonin reuptake inhibitor, it could not improve her disabilities and she came to our clinic. After taking three Kampo medicines, i.e., TJ-9 shosaikoto, TJ-41 hochuekkito, and TY-028 keishikakobokukyoninto, prescribed by our clinic for two weeks, most of her symptoms gradually improved. However, significant general fatigue, headache, pins-and-needles feelings, and brain fog remained, and she could not return to work. Although all results of blood examination, neurological examination, and MRI of the brain and cervical spinal cord were evaluated as normal, the patient’s condition had still not improved a month after COVID-19 infection. At three months post-COVID-19 infection, the patient started an original treatment method for brain fog using neurofeedback for a total of 15 sessions. A medical examination was carried out at each session. After the 1st session, the patient’s headaches no longer occurred, but their concentration was still limited. By the 2nd session, her headaches decreased, and drowsiness improved. By the 3rd session, her headaches disappeared, but the symptoms of cognitive problems such as memory loss and difficulty understanding characters occurred. By the 4th session, feeling like her head was noisy had improved. This state is called a “busy brain” when many things are running through the head and it can be difficult to focus, attention, and think clearly due to being full of thoughts, decreased concentration, anxiety, or stress. By the 5th session, no more headaches had occurred. By the 6th session, her smell disorder and taste disorder completely disappeared. By the 9th session, headaches had reoccurred. By the 10th session, fatigue had become worse. By the 11th session, the patient’s headaches disappeared again. By the 12th session, fatigue and slight anxiety occurred. By the 13th session, the patient’s fatigue continued. By the 14th session, the patient’s overall physical condition had become better, but her concentration was still poor. By the 15th session, the patient’s headaches had completely disappeared and her general condition continued to be good.

Pain and psychological measurements

Pain and pain disability were evaluated using the Short-Form McGill Pain Questionnaire-2 (SF-MPQ-2) [8] and the Pain Disability Assessment Scale (PDAS) [9]. Psychological measurements were completed for anxiety and depression. Anxiety was evaluated based on the Japanese-translated versions of the Pain Anxiety Symptom Scale (PASS-20) [10] and the Hospital Anxiety and Depression Scale (HADS)-A [11]. Depression was estimated using the Japanese-translated versions of the Self-Rating Depression Scale (SDS) [12] and HADS-D [11]. All measurements were carried out before each session and then four days after the 15th session. The percentage improvement for pain, anxiety, and depression was calculated using the following formula: [(value before 1st session) - (value at four days after 15th session]/(value before 1st session) x 100.

Equipment for Z-score neurofeedback

The neurofeedback recorder used was a ProComp Infiniti^TM^ Encoder (Thought Technology Ltd., Montreal, Canada). An all-inclusive software for Z-score neurofeedback was used, called “Z-Score 6 Suite” (Thought Technology Ltd., Montreal, Canada). It contained the program for measurement, “Report - Z-score indices” and the program for treatment, “Z-score index training.” The sensor used was an EEG-ZTM. A TT-EEG 4 Channel Connectivity Kit, NuPrep EEG Skin Prepping Gel, and Ten-20 Conductive EEG Paste were used for the study. All described materials were from Thought Technology Ltd. (Montreal, Canada).

Information for Z-score neurofeedback

Z-score neurofeedback is a type of neurofeedback method that involves real-time comparison of electroencephalograph (EEG) data to the normative database compiled and maintained by Applied Neuroscience Inc. (St. Petersburg, USA). From the raw EEG values, an algorithm in the Z-score software calculates the Z-score deviation based on the NeuroGuide^TM^ normative database. Assessed factors include six metrics: absolute power, relative power, amplitude asymmetry, coherence, phase, and 10 power ratios, including delta/theta, delta/alpha, delta/beta, delta/Hi beta, theta/alpha, theta/beta, theta/Hi beta, alpha/beta, alpha/Hi beta, and beta/Hi beta. Brainwaves are divided into eight wavelengths: delta (1-4 Hz), theta (4-8 Hz), alpha (8-12 Hz), beta (12-25 Hz), Hi beta (25-30 Hz), beta 1 (12-15 Hz), beta 2 (15-18 Hz), and beta 3 (18-25 Hz). Therefore, 248 different output values are applied for the four-channel Z-score neurofeedback treatment. The placements of sensors to the scalp and the earlobe as the ground are aligned with the 10-20 International System [13]. Odd numbers indicate the left hemisphere and even numbers indicate the right hemisphere.

Protocol for Z-score neurofeedback treatment

The Z-score neurofeedback treatment protocol was explained briefly. Informed consent was obtained before beginning the treatment. The method consisted of two rounds. The first round was carried out at C3, C4, T3, and T4, and the second round was carried out at F3, F4, P3, and P4. As four-channel Z-neurofeedback was carried out twice at a time, this method was called “two-by-four 8-channel.”

**Figure 1:**
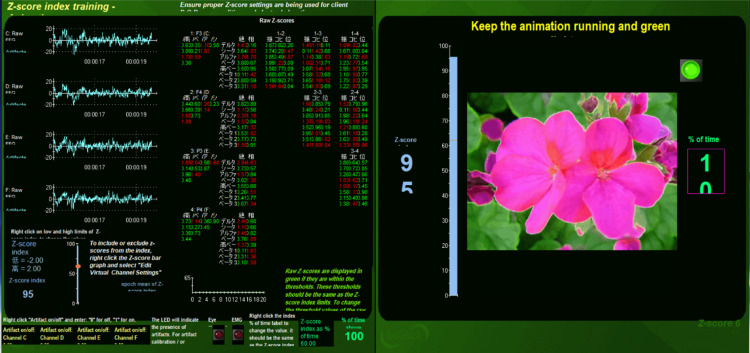
The screen of the program, “Z-score index training.” The upper left shows raw electroencephalograph at F3 (C), F4 (D), P3 (E), and P4 (F). The lower left shows that the threshold of “Z-score index training” is set to between plus 2.0 and minus 2.0 standard deviation and that the Z-score index is set to 95%. The right shows that the animation is running and that the green light is on because the Z-score index is more than 95%. Middle shows each wavelength, six metrics, and 10 power ratios.

For the first round, sensors at C3, C4, T3, and T4 were attached to the patient’s scalp and the eyes were closed; EEG was recorded using “Report - Z-score indices.” Z-score neurofeedback treatment was then examined at C3, C4, T3, and T4 for 10 minutes with the eyes open using “Z-score index training.” All that the patient had to do was to look at the screen during treatment (Figure 1). The threshold should be changed according to each patient’s conditions; an initial setting was plus 2.0 and minus 2.0 standard deviation (SD). From the 13th session, the threshold was changed to plus 1.0 and minus 1.0 SD, as the patient’s symptoms improved. When the raw Z-score fell within the threshold, the number turned green. In contrast, when the raw Z-score fell outside the threshold, the number turned red or yellow. When the average of all raw Z-scores surpassed the Z-score index, images on the screen started moving as a reward (Figure 1). When the average of all raw Z-scores did not surpass the Z-score index, the movement of the images on the screen stopped, and the patient’s brain could not obtain any rewards. The Z-score index was frequently changed depending on the treatment. After treatment, eyes-closed EEGs at C3, C4, T3, and T4 were recorded again. In the second round, the same procedures were performed at F3, F4, P3, and P4. The Z-score neurofeedback treatment typically took about 45 minutes, and a total of 15 sessions with one or two sessions per week were performed.

EEG measurement and analysis

The total values of each channel were evaluated before the 1st session and before the 15th session using “Report - Z-score indices.” The percentage decrease (% decrease) was calculated using this formula: [(total values of each wavelength before 1st session) - (total values of each wavelength before 15th session)]/(total values of each wavelength before 1st session) x 100.

Asymmetry is an imbalance between two homologous sites in the International 10-20 System [13], and this is extremely important and useful for treatments. For example, a high value at the alpha wavelength in the left hemisphere compared with that in the right hemisphere reflects depression, whereas a high value at the higher beta wavelengths in the right hemisphere compared with that in the left hemisphere, or high values at the widespread beta wavelengths, reflects anxiety [14]. As a first step, subtraction of the total values between two homologous sites was calculated at C3-C4, T3-T4, F3-F4, and P3-P4 before the 1st session and before the 15th session. As a second step, each value at each wavelength at each location was measured before the 1st session and before the 15th session, and the percentage increase of each value at each wavelength at C3, C4, T3, T4, F3, F4, P3, and P4 was calculated using this formula: [(each value at each wavelength at each site before 15th session) - (each value at each wavelength at each site before 1st session)]/(each value at each wavelength at each site before 1st session) x 100.

The differences in wavelengths between the sessions were evaluated. The percentage increase of total values at all sites at each wavelength was calculated using this formula: [(total values at all sites at each wavelength after each session) - (total values at all sites at each wavelength before each session)]/(total values at all sites at each wavelength before each session) x 100.

Results of pain and psychological measurements

**Figure 2:**
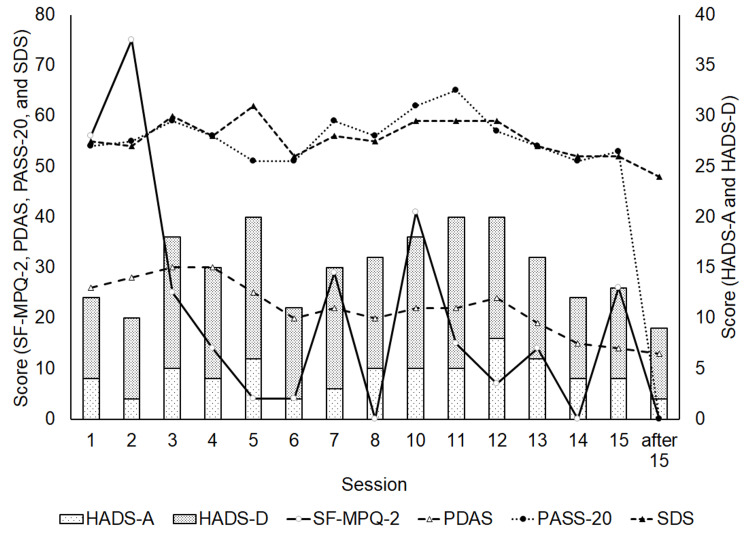
Pain and psychological measurements. SF-MPQ-2: Short-Form McGill Pain Questionnaire-2; PDAS: Pain Disability Assessment Scale; PASS-20: Pain Anxiety Symptom Scale - Japanese translated version; HADS-A: Hospital Anxiety and Depression Scale-Anxiety - Japanese translated version; HADS-D: Hospital Anxiety and Depression Scale-Depression - Japanese translated version; SDS: Self-Rating Depression Scale - Japanese translated version.

Figure 2 shows the results of pain and psychological measurements. The percentage improvements for SF-MPQ-2 and PDAS were 100% and 50%, respectively. These results reveal that pain greatly improved following the Z-score neurofeedback treatment. Regarding anxiety, the percentage improvements for PASS-20 and HADS-A were 100% and 50%, respectively. These results reveal that anxiety also greatly improved following the Z-score neurofeedback treatment. On the contrary, for depression, the percentage improvements for SDS and HADS-D were 12.7% and 12.5%, respectively. These results reveal that depression did not improve substantially.

Results of EEG measurement

Table 1 shows the results of each value at each wavelength at all sites, the total values at all wavelengths at each site before the 1st session and before the 15th session, and the percentage decreases. The percentage decreases at F3, F4, P3, P4, T3, and T4 were positive, whereas the percentage decreases at C3 and C4 were negative. This means that the total values at F3, F4, P3, P4, T3, and T4 decreased after Z-score neurofeedback treatment, whereas the total values at C3 and C4 increased. Asymmetry that the value at the alpha wavelength at C3 was higher than that at C4 before the 1st session indicates depression. On the other hand, it does not mean anxiety that the values at higher beta wavelengths (beta 3 and Hi beta) at C4 were not higher than those at C3 before the 1st session.

**Table 1:** Each value (microvolt) at each wavelength at all sites and the total values at all wavelengths at each site before the 1st session and before the 15th session. The percent (%) decrease is also described.

	Before 1st session								Before 15th session							
	C3	C4	T3	T4	F3	F4	P3	P4	C3	C4	T3	T4	F3	F4	P3	P4
Delta	7.39	6.14	7.08	0	8.7	7.65	7.63	0	6.88	6.54	6.32	0	7.1	5.68	6.03	0
Theta	9.18	7.75	7.83	6.74	9.95	8.73	8.97	8.1	10.6	10.43	9.22	8.34	10.48	8.42	8.56	10.46
Alpha	10.77	9.13	9.27	8.21	12.38	10.79	12.02	12.32	13.25	13.23	11.15	10.8	12.52	11.55	11.47	12.37
Beta	8.39	6.95	8.62	7.12	8.47	7.43	8.55	8.11	7.79	7.44	7.34	6.49	7.48	7.03	7.78	7.15
Hi beta	3.43	2.66	4.33	3.08	3.13	2.53	2.91	2.66	2.68	2.49	2.63	2.17	2.4	2.1	2.41	2.23
Beta 1	4.97	4.2	4.64	4.06	5.04	4.48	5.06	4.77	4.53	4.38	4.14	3.85	4.48	4.09	4.69	4.36
Beta 2	4.48	3.66	4.4	3.64	4.39	3.9	4.6	4.4	4.42	4.17	4.09	3.66	4.19	4.02	4.54	3.95
Beta 3	5.17	4.21	5.74	4.43	5.07	4.35	5.17	4.83	4.78	4.37	4.59	3.84	4.42	4.03	4.46	4.25
Total	53.78	44.7	51.91	51.91	57.13	49.86	54.91	45.19	54.93	53.05	49.48	39.15	53.07	46.92	49.94	44.77
% decrease	-	-	-	-	-	-	-	-	-2.14	-18.68	4.68	24.58	7.11	5.90	9.05	0.93

**Figure 3:**
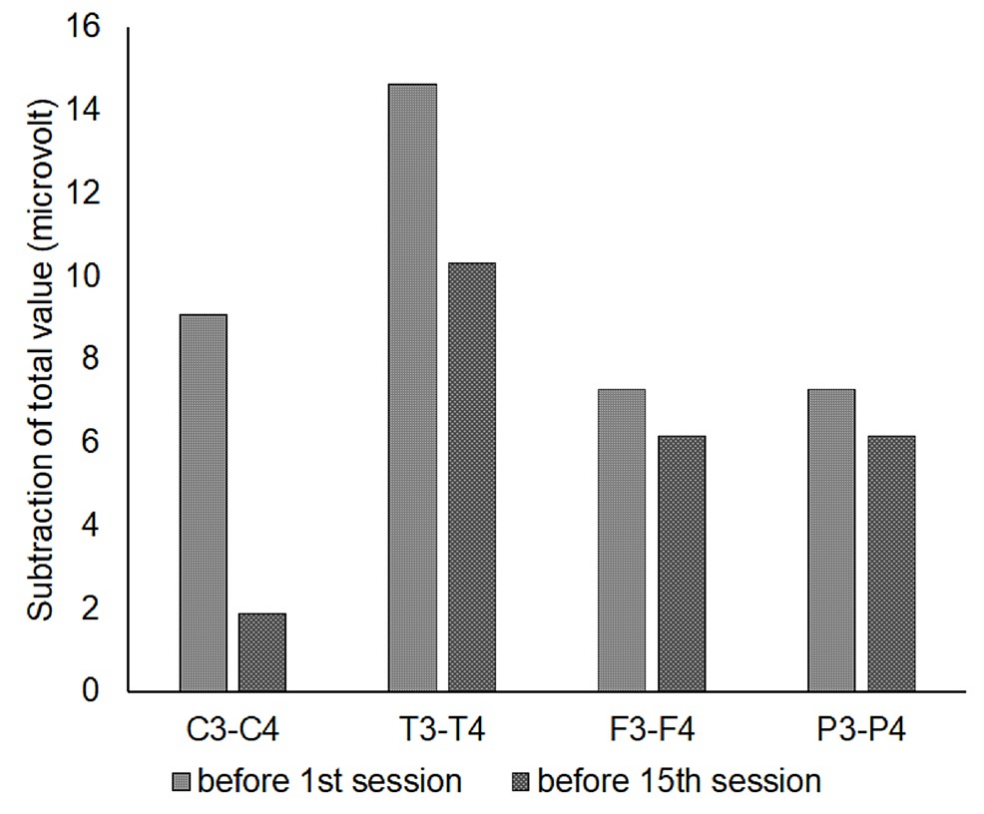
The subtraction of total values (microvolt) between two homologous sites at C3-C4, T3-T4, F3-F4, and P3-P4 before the 1st session and before the 15th session.

Figure 3 shows the subtraction of the total values between two homologous sites before the 1st session and before the 15th session. As all values at all pairs were positive, the values from the left hemisphere were higher than those from the right hemisphere. After the Z-score neurofeedback treatment, the values of subtraction for all pairs got smaller, and C3-C4 was the smallest of all pairs.

**Figure 4:**
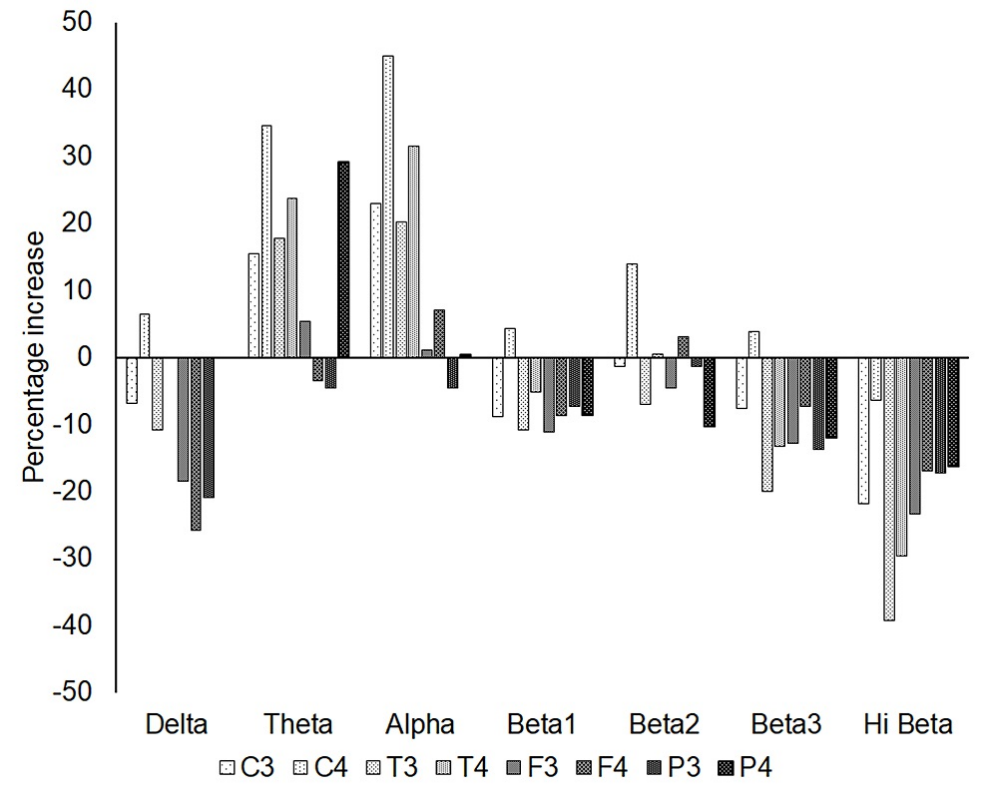
The percentage increase of each value at each wavelength at C3, C4, T3, T4, F3, F4, P3, and P4.

Figure 4 shows the percentage increase for each wavelength at C3, C4, T3, T4, F3, F4, P3, and P4. The theta and alpha wavelengths at almost all sites increased after Z-score neurofeedback treatment, whereas delta, beta 1, beta 2, beta 3, and Hi beta decreased. Moreover, the percentage increase of higher beta frequencies, such as beta 3 and high beta wavelength, showed a greater decrease.

**Figure 5:**
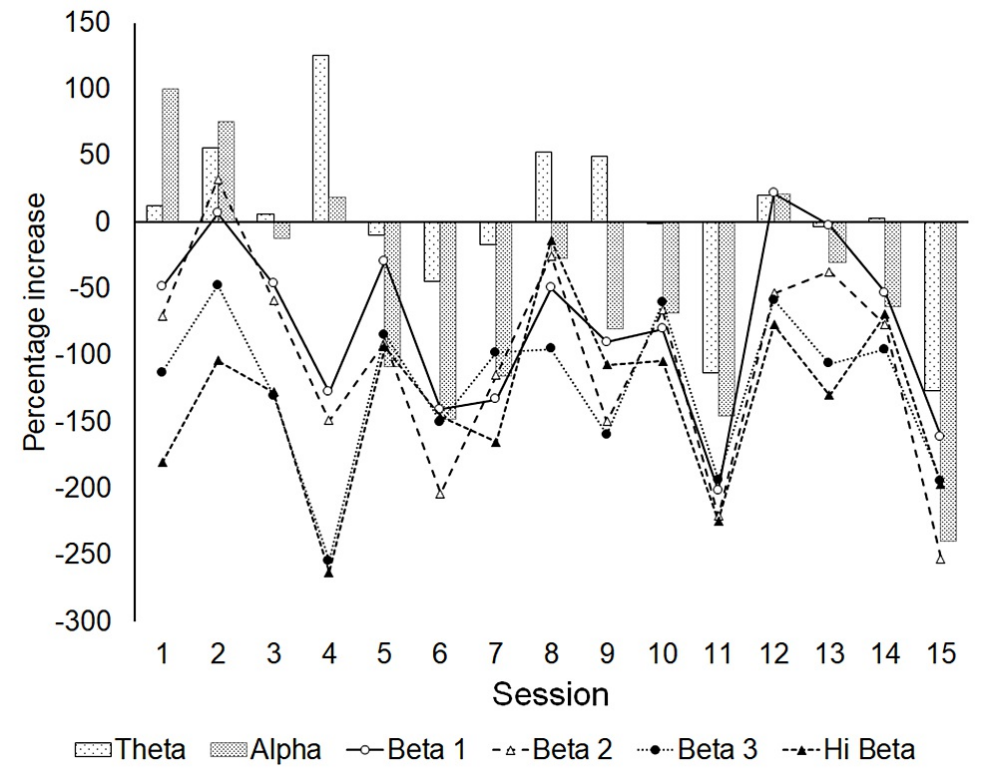
The percentage increase of total values within all sites at each wavelength.

Figure 5 shows the percentage increase of the total values within all sites at each wavelength. The value at the alpha wavelength increased at an early stage of the Z-score neurofeedback treatment, such as at the 1st and 2nd sessions. The value at the theta wavelength increased at the 1st, 2nd, 3rd, and 4th sessions. In addition, the value at the higher beta frequencies such as beta 3 and Hi beta decreased across all sessions. These results are considerably different from the fact that no change of the value at wavelength occurred at a small number of sessions in the field of neurofeedback [15]; this is discussed in the Discussion section.

## Discussion

Although attempts have been made worldwide to develop treatments for brain fog, no single, highly effective method has been established. Neurofeedback is an advanced technique that enables patients to recover from brain disorders, including anxiety, depression, insomnia, headaches, and pain [3]. Neurofeedback also enables patients to improve cognitive functions, such as processing speed, and executive functions, including attention, planning, organization, problem-solving, and performance [4]. Neurofeedback is a highly promising treatment method for brain fog because it is effective for “chemofog” and “chemobrain” [7], which have similar symptoms to that of brain fog. By regulating values at certain wavelengths upward or downward, brain function can be improved and problematic symptoms can be reduced. If the value at a certain wavelength is moved in a desirable direction using the neurofeedback protocol, positive visual or auditory rewards are given. If not, no reward is given. In this way, patients can learn how to obtain rewards. Their brain function gradually improves, and problematic symptoms are reduced little by little [16].

Neurofeedback has one evaluation method and two treatment methods. Quantitative EEG (QEEG) is the most well-known evaluating method, and it can handle all channels of the 10-20 International System [13] at once. Single-channel neurofeedback is a standard treatment method that handles only one impaired site based on the results of a preceding QEEG. If a QEEG cannot be used, a symptom-specific site is selected. However, if more than two sites are needed for treatment, multiple time operations will be required at the same time, which presents an issue for single-channel neurofeedback. In the case of brain fog, multiple sites of the brain are assumed to be impaired, so single-channel neurofeedback needs multiple time operations at once, and this takes a relatively long time. Therefore, single-channel neurofeedback is not desirable for the treatment of brain fog. Z-score neurofeedback is a treatment method that can handle multiple sites at one time. Thus, Z-score neurofeedback is believed to be promising for the treatment of brain fog. The currently used all-inclusive software, “Z-Score 6 Suite,” can handle up to four channels at once. Other advantages of Z-score neurofeedback are that it is simple and quick to learn and it can be applied to many clinical disorders [17].

Z-score neurofeedback has two user-defined setting indices: threshold and Z-score index. The threshold is between the lower limit and upper limit, and it is changed depending on the condition of the patient and the progress of their treatment. In this current report, the starting threshold was plus and minus 2.0 SD. From the 13th session, the threshold was changed to plus and minus 1.0 SD because the symptoms of the patient had improved. The Z-score index is the lower limit of the average of all raw Z-scores within the threshold. When the average at a given moment is above the Z-score index, rewards are given. The Z-score index should be frequently changed during the session depending on the status of the treatment.

Selection of the treated sites of the brain is extremely important for the efficacy of neurofeedback. F3, F4, P3, and P4 represent the left frontal lobe, right frontal lobe, left parietal lobe, and right parietal lobe, respectively. Balancing these four sites with Z-score neurofeedback promotes cognitive regulation and mood stability. Therefore, if patients present with cognitive issues, anxiety, or depression, Z-score neurofeedback treatment at F3, F4, P3, and P4 is preferable [17]. Another promising combination of sites is C3, C4, T3, and T4 because they represent the left central lobe, right central lobe, left temporal lobe, and right temporal lobe, respectively. Balancing these four sites promotes emotional regulation. If patients present with emotional instability or sensory imbalance, Z-score neurofeedback treatment at C3, C4, T3, and T4 is desirable [17]. Furthermore, each site has a site-specific function [17]. For example, C3 and C4 are in proximity to the insula, which helps to regulate mood, pain, and social well-being [17]. Because C3 and C4 are also in the sensorimotor strip (SMR), fine motor skills are regulated at C3 and C4. T3 and T4 are near the hippocampus, which assists in memory processing [17]. As pathophysiological findings of brain fog indicate direct viral neuronal damage, neuroinflammation, rupture of the blood-brain barrier, microvasculitis, and hypoxia [1], a widespread area of the brain is assumed to be damaged in brain fog. Therefore, treatment at as many sites as possible and the inclusion of function-specific sites are highly preferable. As our original method can handle up to eight channels, it is highly promising as a new treatment method for brain fog. In this case report, the patient suffered not only from brain fog, but also from chronic pain, depression, and anxiety; hence, we developed the total eight-channel method for this reason.

Table 1 shows that the total values at F3, F4, P3, P4, T3, and T4 decreased after Z-score neurofeedback treatments, whereas those at C3 and C4 increased. Furthermore, the percentage decrease at C4 was the lowest of the three. This means that the impairments at C3 and C4 occurred because of brain fog and were improved by Z-score neurofeedback, with C4 being the most affected and improved site.

Figure 3 shows that the value of subtraction at all homologous site pairs gets smaller after Z-score neurofeedback treatment. This reveals that brain fog increases the difference between homologous site pairs and that Z-score neurofeedback treatment can recover this difference. Moreover, Figure 3 also shows that the value of subtraction at C3-C4 after Z-score neurofeedback treatment was the smallest. This result indicates that C3 and C4 would be the most easily influenced sites by Z-score neurofeedback treatment. Interestingly, C3 and C4 are reported to be good indicators for depression and anxiety [14].

The percentage increase at theta and alpha wavelengths at almost all sites increased with Z-score neurofeedback treatment, whereas delta, beta 1, beta 2, beta 3, and Hi beta at almost all sites decreased. In addition, the higher frequencies of beta wavelengths (beta 3 and Hi beta) showed a greater decrease (Figure 4). As high values at the alpha wavelength mean a relaxed or calm state, and high values at the higher beta wavelengths mean excessive stress, anxiety, high arousal, and an inability to relax [18], the increased value at the alpha wavelength and the decreased values at the higher beta wavelengths greatly align with the decreases in PASS-20 and HADS-A (anxiety), and SDS and HADS-D (depression) (Figure 2).

Figure 2 also indicates that improvements in pain and anxiety were obtained, whereas no substantial improvements were obtained for depression. A fairly high value at the alpha wavelength in the left hemisphere compared with that of the right hemisphere reflects depression, whereas fairly high values at the higher beta wavelengths in the right hemisphere compared with those of the left hemisphere, or high values at the widespread beta wavelengths, reflect anxiety [14]. As the value at the alpha wavelength at C3 before the 1st session was higher than that at C4, depression occurred due to brain fog. The actual value of the difference between the value at the alpha wavelength at C3 and that at C4 before the 1st session was 1.64. After Z-score neurofeedback treatment, this difference became 0.02. In contrast, anxiety did not occur because the value at the higher beta wavelengths at C4 before the 1st session was not higher than that at C3 (Table 1). Notably, the patient was in a state of depression under the influence of brain fog, and therefore, the improvement in depression was not substantial.

Many reports describe that an average of 30-40 sessions are usually needed for neurofeedback treatment to be effective for common disorders, including attention deficit disorder (ADD)/attention deficit hyperactivity disorder (ADHD), anxiety, depression, and insomnia [19], and that patients can start to notice some changes such as improved sleep, more energy or motivation, improved concentration and focus, and a greater feeling of relaxation and calmness after five to six sessions. In addition, the benefits from neurofeedback usually start to last for more than one to two days, and they can last up to one week or more after 20 or more sessions are completed [15]. Figure 5 shows that the values at the theta and alpha wavelengths increased after the 1st session and 2nd session. As the alpha wavelength indicates relaxation and calmness [18], an increased value at the alpha wavelength was highly preferable for recovery from brain fog. Similarly, the values at the higher beta wavelengths such as beta 3 and Hi beta decreased at all sessions. As a fairly high value at higher beta wavelengths indicates excessive stress, anxiety, high arousal, and an inability to relax [18], decreased values at higher beta wavelengths were greatly preferable. These findings are far from common understanding and suggest great potential for Z-score neurofeedback treatment for brain fog.

Neurofeedback has been used for over 50 years, is generally considered to be safe, and does not produce harmful side effects [20]. Although neurofeedback treatments occasionally cause some temporary reactions, such as fatigue, headaches, lightheadedness, dizziness, irritability, moodiness, weeping, insomnia, agitation, difficulties with focus, and anxiety, they usually fade away after one to two days [16]. In this case report, no side effects occurred.

## Conclusions

As our first patient successfully recovered, an original neurofeedback treatment method for brain fog using the Z-score neurofeedback technique is believed to be effective in improving the symptoms of brain fog from COVID-19. The fact that the percentage improvements about anxiety improved more than those about depression for this patient aligned with the fact that the patient was in a higher state of depression than anxiety, which was based on the results from psychological measurements and EEG findings. C4 was evaluated to be the site most affected by brain fog and the most improved site after treatment, as the percentage decrease at C4 was the lowest across all sites, and the subtraction of total values at C3-C4 before the 15th session was the smallest of all pairs. The finding that the percentage increase at alpha wavelengths increased at almost all sites and beta wavelengths decreased at almost all sites after Z-score neurofeedback treatment corresponded with the results of psychological measurements. The increased values at the theta and alpha wavelengths after the 1st and 2nd sessions and the decreased values at the higher beta wavelengths were shown at all sessions, although previous studies report that 30-40 sessions on average are usually needed for effective neurofeedback treatments for ADD/ADHD, anxiety, depression, and insomnia.

## References

[1] Ellul MA, Benjamin L, Singh B (2020). Neurological associations of COVID-19. Lancet Neurol.

[2] Callan C, Ladds E, Husain L, Pattinson K, Greenhalgh T (2022). 'I can't cope with multiple inputs': a qualitative study of the lived experience of 'brain fog' after COVID-19. BMJ Open.

[3] Tan G, Shaffer F, Lyle R, Teo I (2016). Evidence-Based Practice in Biofeedback & Neurofeedback, 3rd Edition. Association for Applied Psychophysiology and Biofeedback, Wheat Ridge, USA.

[4] Angelakis E, Stathopoulou S, Frymiare JL, Green DL, Lubar JF, Kounios J (2007). EEG neurofeedback: a brief overview and an example of peak alpha frequency training for cognitive enhancement in the elderly. Clin Neuropsychol.

[5] Theoharides TC, Cholevas C, Polyzoidis K, Politis A (2021). Long-COVID syndrome-associated brain fog and chemofog: luteolin to the rescue. Biofactors.

[6] Inagaki M, Yoshikawa E, Matsuoka Y (2007). Smaller regional volumes of brain gray and white matter demonstrated in breast cancer survivors exposed to adjuvant chemotherapy. Cancer.

[7] Alvarez J, Meyer FL, Granoff DL, Lundy A (2013). The effect of EEG biofeedback on reducing postcancer cognitive impairment. Integr Cancer Ther.

[8] Maruo T, Nakae A, Maeda L, Takahashi K (2013). Translation and reliability and validity of a Japanese version of the revised Short-Form McGill Pain Questionnaire (SF-MPQ-2). (Article in Japanese). Pain Res.

[9] Yamashiro K, Arimura T, Iwaki R, Jensen MP, Kubo C, Hosoi M (2011). A multidimensional measure of pain interference: reliability and validity of the pain disability assessment scale. Clin J Pain.

[10] McCracken LM, Dhingra L (2002). A short version of the Pain Anxiety Symptoms Scale (PASS-20): preliminary development and validity. Pain Res Manag.

[11] Annunziata MA, Muzzatti B, Bidoli E (2020). Hospital Anxiety and Depression Scale (HADS) accuracy in cancer patients. Support Care Cancer.

[12] Zung WW (1965). A self-rating depression scale. Arch Gen Psychiatry.

[13] Demos JN (2019). Electrode placements. Getting Started With EEG Neurofeedback, Second Edition.

[14] Demos JN (2019). Matching EEG signatures to common symptoms and disorders. Getting Started With EEG Neurofeedback, Second Edition.

[15] Longo R (2021). What is quantitative electroencephalography (QEEG) brain mapping?. A Consumer's Guide to Understanding QEEG Brain Mapping and Neurofeedback Training.

[16] Demos JN (2019). Thresholds: advanced theory of protocol operation. Getting Started With EEG Neurofeedback, Second Edition.

[17] Demos JN (2019). Z-score training concepts and concerns. Getting Started With EEG Neurofeedback, Second Edition.

[18] Longo R (2021). Chapter One. What is quantitative electroencephalography (QEEG) brain mapping?. A Consumer's Guide to Understanding QEEG Brain Mapping and Neurofeedback Training.

[19] Longo R (2021). Introduction. A Consumer's Guide to Understanding QEEG Brain Mapping and Neurofeedback Training.

[20] Longo R (2021). Preparing for neurofeedback. A Consumer's Guide to Understanding QEEG Brain Mapping and Neurofeedback Training.

